# A multi-country comparison of stochastic models of breast cancer mortality with P-splines smoothing approach

**DOI:** 10.1186/s12874-020-01187-5

**Published:** 2020-12-09

**Authors:** Sumaira Mubarik, Ying Hu, Chuanhua Yu

**Affiliations:** 1 Department of Epidemiology and Biostatistics, School of Health Sciences, Wuhan University, 185 Donghu Road, Wuhan, 430071 Hubei China; 2 Global Health Institute, Wuhan University, Wuhan, 430071 Hubei China

**Keywords:** Breast cancer mortality, Goodness of fits, Lee carter model, Multivariate Diebold-Marino-test, P-splines

## Abstract

**Background:**

Precise predictions of incidence and mortality rates due to breast cancer (BC) are required for planning of public health programs as well as for clinical services. A number of approaches has been established for prediction of mortality using stochastic models. The performance of these models intensely depends on different patterns shown by mortality data in different countries.

**Methods:**

The BC mortality data is retrieved from the Global burden of disease (GBD) study 2017 database. This study include BC mortality rates from 1990 to 2017, with ages 20 to 80+ years old women, for different Asian countries. Our study extend the current literature on Asian BC mortality data, on both the number of considered stochastic mortality models and their rigorous evaluation using multivariate Diebold-Marino test and by range of graphical analysis for multiple countries.

**Results:**

Study findings reveal that stochastic smoothed mortality models based on functional data analysis generally outperform on quadratic structure of BC mortality rates than the other lee-carter models, both in term of goodness of fit and on forecast accuracy. Besides, smoothed lee carter (SLC) model outperform the functional demographic model (FDM) in case of symmetric structure of BC mortality rates, and provides almost comparable results to FDM in within and outside data forecast accuracy for heterogeneous set of BC mortality rates.

**Conclusion:**

Considering the SLC model in comparison to the other can be obliging to forecast BC mortality and life expectancy at birth, since it provides even better results in some cases. In the current situation, we can assume that there is no single model, which can truly outperform all the others on every population. Therefore, we also suggest generating BC mortality forecasts using multiple models rather than relying upon any single model.

**Supplementary Information:**

The online version contains supplementary material available at 10.1186/s12874-020-01187-5.

## Background

The modeling and projections of future cancer related incidence and mortality rates are essential for development of public health programs and clinical amenities [1]. Breast cancer (BC) ranked among the top global burden of diseases, and it threaten the health all over the world. Among Asian women, the BC is consider the second leading cause of cancer related morbidity and mortality. According to previous studies, the BC among Asian women constitute approximately 40% of all BC diagnosed worldwide [2–4]. Asia has much higher mortality to incidence ratio of BC than western countries [2, 3]. A study conducted on BC differences in Asian regions, reported that BC increasing among Asian women. Cause of this increasing rate are associated with higher prevalence of BC risk factors like, delayed childbirth, increased obesity [5]. Other risk factors that may contribute to increase mortality risk due to BC are include aging process, high body mass index, alcohol consumption and low physical activity [2–4, 6, 7]. Though, a number of cumulative exposures contribute to raise the BC mortality rates, therefore it is expected that these rates follow a smooth curvilinear pattern and future mortality rates are predictable based on these pattern. The rapid change pattern of BC rates over the historical time is remain a challenge for its prediction. The Asian countries selected in this study are suffering from increase BC burden and also having similar circumstances related to over population, poverty, socio-cultural background, and insufficient access to diagnosis, advanced screening and proper treatment [4]. Moreover, due to lack of proper statistical registries system in the developing countries, statisticians always encounter the problem of insufficient and unsatisfying Asian demographic and disease registration data sets. This data deficiency raise the problems when  it used in stochastic mortality models. Solving those problems bring lots of research passion in this field. In light of limited data access combined with poor quality, less technically refined methods are available for Asian countries compared with developed countries. One of the methods which could be applied on Asian data is the stochastic population approach. This approach has been used in various studies to generate the future mortality forecasts for different western countries [8].

Till now, variety of forecasting techniques for prediction of mortality trends have been tested and proposed consistently [9, 10]. In this regard, the work of Lee and Carter [11] is incontrovertible contribution in literature of mortality forecasting. Their contributions based on generalization of different stochastic mortality models and its extended versions. The basic Lee-Carter (LC) model uses age and time parameter to estimate the future mortality trends by using principal component approach. This initial model now considered a milestone in the stochastic modeling and forecasting of trends in mortality data. After this, various extensions and modifications were proposed. These extensions vary in number of fundamental features, for example, smoothness assumption, causes of randomness, different estimation methods and addition of cohort effects. Now these methodologies has become widely used to attain a broader interpretation and to capture the main structures of vigorous of mortality intensity [12–17]. However, in context of different stochastic mortality models comparisons, many studies confirm that there is no single model among them which clearly dominates the others according to measured evaluation standards [18–20]. Lee and Tuljapurkar [8] proposed a new method in case of few observations at uneven intervals, and they applied it to China and South Korea data. Hyndman and Ullah [15] developed a more general method by treating the underlying demographic process as functional data, employing the functional principal components to extract more than one explaining components and providing robust estimation and forecast. Previously, functional demographic models on breast cancer mortality data has been used to estimate the future trends in breast cancer mortality for United State and England-Wales [21].

This study apply the three stochastic mortality models belong to family of generalized LC with smoothing p-splines approach to four Asian countries’ data sets, evaluate the performance of these three methods, compare the similarities of different Asian regions and accordingly propose potential improvements. The models that apply in current study named as smoothedleecarter (SLC) model, Functional demographic model (FDM) and Booth-Maindonald-Smith (BMS) model. Though, the prediction of mortality for future by different stochastic methods have been extensively reviewed elsewhere [22, 23]. This paper covers in-sample and out-sample evaluation of different stochastic smoothed mortality methods by applying them to BC mortality of four Asian countries.

**Fig. 1:**
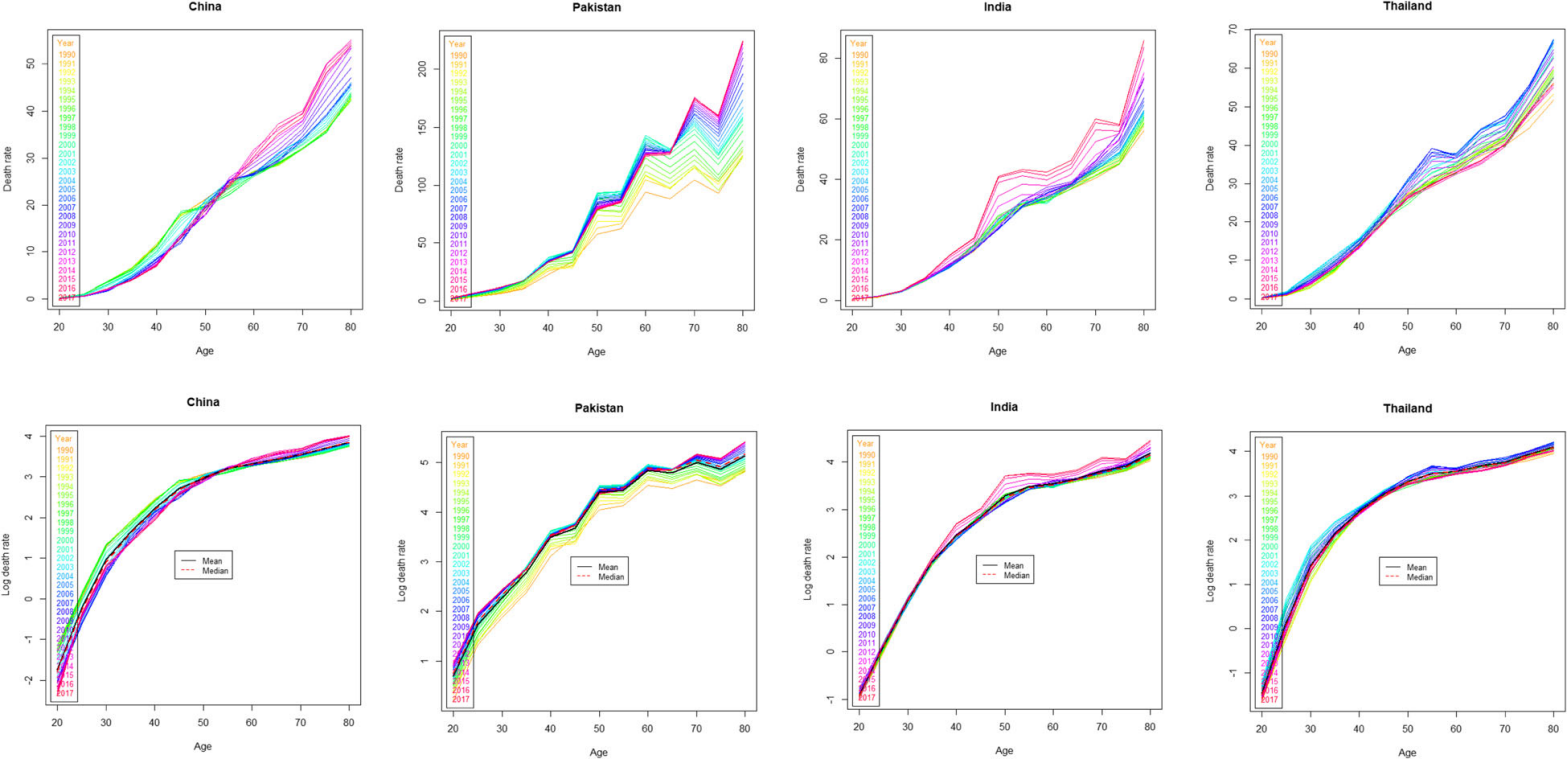
Females breast cancer death rates (per, 100,000) among four countries, 1990–2017, Top horizontal panel = actual death rates, Bottom panel = log death rates

Reference to current study data sets, the parameter estimation of LC models is based on least square method using singular value decomposition (SVD) algorithm of the matrix of the log age-specific observed BC death rates. The BC mortality data in form of death counts and exposures to risk have to fill a rectangular matrix. Hereafter, it will be denoted as *M*_*x*, *t*_ that is observed BC mortality rates at age x during calendar year t. it is achieved by the ratio between the number of deaths at age x during year t, (*D*_*x*, *t*_), from an exposure to risk, that is, the number of person years from which *D*_*x*, *t*_ happened (*E*_*x*, *t*_). As regards the Asian women population data set on the basis of the BC mortality rates from 20 to 80+ year of age, presented by countries and individual year, it shows curvilinear mortality behavior and suggest the smoothing is suitable for this data (see Fig. 1). We can see the random variations in the data, especially for ages below 50, where the reductions in the BC mortality rates are solid. Besides, we can also observe the BC mortality irregularities for older ages. Therefore, the model estimation on four Asian countries’ data set follow the smoothing technique for model fitting to control the random variations in the data, otherwise we could not get the resulting BC mortality rates much reliable.

The aim of current study based in achieving the following specific objectives: (a) To estimate the BC mortality trends by using three stochastic mortality models with smoothing P-splines approach for four Asian countries’ data sets. (b) To evaluate both the goodness of fit and predictive ability (In-sample and Out-sample) of the three models using advance quantitative forecast accuracy test as well as by range of graphical display on each Asian country’s data set. To the best of our knowledge, this is the first study to extend the current literature on Asian mortality data due to breast cancer, on both the number of considered stochastic mortality models and their rigorous evaluation using multivariate Diebold-Marino test and by range of graphical analysis for multiple countries under consideration. Our research to some extent validates the existing results but also presents a more comprehensive view. The rest of the current study is organized in the following sections. The methodology is addressed in section 2 and results of empirical analysis is presented in section 3. Discussion and conclusion is merged in Section 4.

## Methods

In this section, analysis methods of BC mortality modeling is discussed. P-splines approach for Smoothing BC mortality rates were employed first. Then, three stochastic mortality models were fitted to the smoothed BC mortality data. Goodness of fits tests included graphical displays and multivariate Diebold–Mariano (DM) test was used to evaluate the model performance.

### Smoothing data using P-splines approach

The violation of homogeneity assumption due to presence of outliers in mortality data may often under or overestimate the actual mortality estimates. Particularly, the mortality rates in older ages have high variability because of small number of cases in the population. This high variability can create the problems in estimation of mortality rates for older ages. Therefore, use of smoothing techniques for such a data can avoid this deficiency of facts. Otherwise, the heavy difference at older ages influences the fitting of mortality models and can over or under fit the model [24]. To overcome this situation various studies suggested the Penalized splines which also known P-splines is now widely used method of smoothing in stochastic mortality models and generalized linear models [9, 25–27]. Concerning to its application, the following main features of the methodology is adopted: Firstly, the B-splines used as the basis for the regression modeling; secondly, the regression coefficients with different penalties is used for modifying the log-likelihood. In line with the previous literature, the intensity of mortality is decomposed as follow: [26, 28].

1 $$ \mathit{\ln}{\mu}_x(t)=\sum \limits_{i,j}{\Theta}_{i,j}{B}_{i,j}\left(x,t\right) $$

The two-dimensional B-splines *B*_*i*, *j*_ for specific age and calendar year x and t respectively with regularly spaced knots is used. The parameters Θ_*i*, *j*_ ’s, to be estimated based on the data set. As suggested by a study, the penalty can be used based on finite differences of the coefficients of the adjacent B-spline to limit the influence of the knots on the fitted value and this approach is called P-spline [25]. The calculation of penalties for both dimensions age x and calendar year t, depend on sums of following components (Θ_*i*, *j*_ − 2Θ_*i* − 1, *j*_ + Θ_*i* − 2, *j*_)^2^. The selection of weight coefficients to attach with each penalty should depend on historical data. According to some studies, the P-splines are less apparent to the actuaries, particularly due to the selection of penalty parallels to the assessment of the future pattern of mortality. In order to smooth the mortality data, a study has reported the limits of using a penalized spline. As the penalty depends on the parameterization, so smoothed value is vary with respect to its choice [10]. Its solution is suggested in a study, to consider a direct smoothing and interchange the penalized differences in the contiguous coefficient with the penalized differences in the contiguous fitted values [28]. Recommended by a study, it is better to smoothing the data initial, rather than smoothing the fitted values. In this way, it become more convenient to impose the monotonic restriction on the smoothing data more simply [15].

### The Lee–Carter (LC) model

The Lee-Carter (LC) model is backbone of all methodologies that used for mortality projection and it is key component of all actuarial literature related to mortality forecasting. The LC method (Lee and Carter [11]) for forecasting mortality rates uses principal components analysis to decompose the age-time matrix of central death or mortality rates into a linear combination of age and time parameters. The parameter of time is used in forecasting. LC has produced numerous variants and extensions. The two main variants of LC are Lee Miller (LM) and Booth-Maindonald-Smith (BMS) presented by lee and miller [29], and Booth et al. [12] respectively. These variants are collectively referred to as “LC methods”. A major extension of this approach uses functional data analysis. Which has presented in next sections. It further extended in various combinations of options that study variation in different dimension like age, period or cohort, and these are famous as “HU methods. Detail description of methods and extensions can be found elsewhere [23, 30].

With reference to the present study data set, the following Eq. (2) shows the LC model for BC mortality data. The logarithm of the observed BC mortality rates given for age x and year t is denoted as *M*_*x*, *t*_.The sum of an age-specific component *α*_*x*_ represent the independent of time parameter. Another component that is product of a time-varying parameter *k*_*t*_, and an age-specific component *β*_*x*_, reflecting the general level of BC mortality and change of BC mortality at each age x with changes in general level of BC mortality respectively.

2 $$ \ln \left({M}_{x,t}\right)={\alpha}_x+{\beta}_x{k}_t+{\varepsilon}_{x,t} $$

The error term is denoted as *ε*_*x*, *t*_ and it is assume that it follow homoscedastic and normality assumption. In order to find a least squares solution to the Eq. (2) the SVD method is used because of unavailability of regressors on right-hand side. To find the unique solution of parameter of Eq. (2) the following set of constrains is imposed on parameters of Eq. (2), firstly, the sum of the age specific coefficients is equal to one i.e. ∑*β*_*x*_ = 1 and secondly, the sum of the time varying parameter is equal to zero i.e. ∑*k*_*t*_ = 0. In order to forecast BC mortality by using the LC model, following two procedures were adopted in current study. First procedure is to estimate parameters *α*_*x*_, *β*_*x*_ and *k*_*t*_ using historical BC mortality data. Second procedure is to estimated time-dependent parameter *k*_*t*_. Model structure is based on a stochastic process which is follow autoregressive integrated moving average (ARIMA p, d, q) model scheme. It is commonly known as the standard Box and Jenkins methodology [31, 32]. Finally, the fitted ARIMA model was used to extrapolate *k*_*t*_ and to get a forecast of future BC mortality rates and then from these forecasts future life expectancy was deriven to evaluate the model.

### Booth-Maindonald-Smith (BMS) model

In LC model the age-specific component *β*_*x*_, which can be defined in terms ${\beta}_x^{(1)}$ and ${\beta}_x^{(2)}$ are assumed to be constant over time and time varying parameter ${k}_t^{(2)}$ is assumed to be linear over time. Booth, Maindonald and Smith [12] changed these assumption in their study. In line with the prior literature, the BMS model was built by extended the LC model. The extension of model based on inclusion of more interaction terms between age x and year t. Model fitting period was restricting by improving the assumptions of homoscedastic and linearity of *β*_*x*_ and *k*_*t*_ respectively. However, in LC model only first terms of the SVD is used. While, BMS model can used “n” terms that allow the model to include second and higher order terms as well [22]. According to a study, any systematic variation in the residuals from fitting only the first term would be captured by the second and higher order terms [12]. With reference to current study data set, the BMS model can be presented as:

3 $$ \ln \left({M}_{x,t}\right)={\alpha}_x^0+{\sum}_{i=1}^n{\beta}_x^{(i)}{k}_t^{(i)}+{\varepsilon}_{x,t} $$

Where *M*_*x*, *t*_ is the BC mortality rate for age x in calendar year t. ${\alpha}_x^0$ is the intercept of the model which represent the effect of age-specific parameter. ${\beta}_x^{(i)}$ represent the “age interaction” parameter at age x. While, ${k}_t^{(i)}$ is the “time interaction” parameter that indicate the value in year t. The residual term *ε*_*x*, *t*_ shows the value of error at age x and year t and approximation rank is denoted by n.

The purpose of inclusion of more interaction terms in the BMS model is to improve the model fit on data and to increase its capacity to explain more unexplained variations that could not account in LC model. The BMS model applied by a study on Australian data over the period 1907 to 1999. The study results reported the higher forecasted life expectancies and smaller forecast error by BMS model relative to the LC model. Another study also claim the more accurate forecasts and use of shorter fitting period by BMS model than the LC model [33, 34].

### Functional Demographic Model (FDM)

Hyndman and Ullah [15] presented the modified version of the LC model, which is known as Functional Demographic Model (FDM). This methodology is based on combination of functional data analysis and nonparametric smoothing and robust statistics and it was proposed to forecast the age-specific mortality rates. Particularly, this approach allows for smooth functions of age, resulting the robust to outliers and offers a modeling framework convenient to fit to restrictions and other evidence. The methodology background of FDM based on generalization of LC method. In context of our study data set, let *y*_*t*_(*x*) represent the log of the observed BC mortality rates for specific age x and calendar year t i.e. *y*_*t*_(*x*) = ln(*M*_*x*, *t*_) and we assume that there are underlying ${\mathbbm{L}}_2$ continuous and smooth functions {*f*_*t*_ (*x*)} such that

4 $$ {y}_t\left({x}_i\right)={f}_t\left({x}_i\right)+{\sigma}_t\left({x}_i\right){\varepsilon}_{t,i} $$

where, *ε*_*t*, *i*_ is error term which is assume to be independent identically distributed (i.i.d) standard normal variable, and quantity of noise that vary with age *x* is denoted by *σ*_*t*_(*x*_*i*_). The forecasting of log observed BC mortality rates *y*_*t*_(*x*) founded through following steps:

- Step-1. First of all smoothed data set series was generated for each time t using penalized regression splines. The functions *f*_*t*_(*x*) for *x* ∈ [*x*_1_, *x*_*p*_] from *ℝ*^2^, where *ℝ*^2^ = {(*x*_*i*_, *y*_*t*_(*x*_*i*_) ∣ (*i*, *t*) ∈ *ℕ*_*p*_ × *ℕ*_*n*_}. The functions *f*_*t*_(*x*) for i = 1,2,...,p was estimated for each time t i.e. t = 1,2, … n by applying a nonparametric smoothing with constraint. It was assume that the function *f*_*t*_(*x*) is monotonically increasing for x ≥ c for some c (say 65 years), where c is reasonable threshold for mortality data. The estimated curves has less noise at older ages by imposing this condition (see Hyndman and Ullah [15], for more details).
- Step 2. The smoothed curves *f*_*t*_(*x*) generated in above step-1 was decomposed by using the following basis function expansion [35].

5 $$ {f}_t(x)=\mu (x)+{\sum}_{k=1}^K{\beta}_{t,\mathrm{k}}{\phi}_k(x)+{e}_t(x),\kern0.5em x\in \left[{x}_1,{x}_p\right] $$

where *μ*(*x*) is a locational measurement (median curve) of *f*_*t*_(*x*), while Eigen functions *ϕ*_*k*_(*x*) shows the main regions of variation or kth principal component function; { *β*_*t*, *k*_ } are the coefficients (or corresponding principal component scores) which are uncorrelated by construction and k is the number of basis functions with k < n; Hyndman and Booth [36] found that k = 6 is sufficient to capture a substantial amount of variance in the data; *e*_*t*_(*x*) is the error function which ∼ N (0,var.(x)).

- Step 3. By using Eq. (5), a univariate time series model to each principal component score { *β*_*t*, *k*_ } was applied to obtain their future values.
- Step 4. Based on fitted time series models obtained in step 3, the coefficients { *β*_*t*, *k*_ }, k = 1,2,...,K, are forecasted for t = n + 1,...,n + h.
- Step 5. The forecasted coefficients attained in the step 4 was applied in Eq. (5) to get the *f*_*t*_(*x*). Then *y*_*t*_(*x*) was forecasted from Eq. (4). In simple word, the *y*_*t*_(*x*_*i*_) can be simplify by combining (4) and (5) and resulting equation was obtained as (6):

6 $$ {y}_t\left({x}_i\right)=\mu \left({x}_i\right)+{\sum}_{k=1}^K{\beta}_{t,k}{\phi}_k\left({x}_i\right)+{e}_t\left({x}_i\right)+{\sigma}_t\left({x}_i\right){\varepsilon}_{t,i} $$

Specifically, the h-steps ahead forecasts of *y*_*n* + *h*_(*x*) can be obtained by using following formula (7):

7 $$ {y}_{n+h}(x)=E\left[{y}_{n+h}(x)\left|\mathrm{I},\Phi \right.\right]=\hat{\mu}(x)+{\sum}_{k=1}^K{\hat{\beta}}_{n,k,h}\kern0.5em {\hat{\phi}}_k(x) $$

In above Eq. (7), the observed data is denoted as I = {*y*_*t*_(*x*_*i*_); t = 1,2,...,n; i = 1,2,...,p,} and Φ is the set of basis functions (or estimated set of functional principal components). The ${\hat{\beta}}_{n,k,h}$ corresponds to the h-step ahead forecast of *β*_*n* + *h*, *k*_ having been estimated time series ${\hat{\beta}}_{1,k,}\dots {\hat{\beta}}_{n,k,}$ using Eq. 7.

- Step 6. Lastly, the variance of error terms in Eqs. (4) and (5) can be calculated to determine confidence intervals for mortality projections. In particular, the forecast variance can be derived from Eq. (6) which is explained in prior studies [10, 30]. .Due to complex theoretical calculation of interval forecast with the fitted model, various studies suggested this equation as useful tool for interval forecast calculation [21, 37, 38].

### Forecast accuracy measure

In order to measure the forecast accuracy, the difference between actual and corresponding forecast series, indicated as mean absolute forecast error (MAFE) is calculated by given formula,

8 $$ {MAFE}_{\upxi}=\frac{1}{J}\ {\sum}_{j=1}^J\left|{y}_{\mathrm{n}+\upxi \left({x}_j\right)}-{\hat{y}}_{\mathrm{n}+\upxi \left({x}_j\right)}\right| $$

In Eq. (8) the term ${y}_{\mathrm{n}+\upxi \left({x}_j\right)}$ is the actual observed sample values for the j_th_ age and ξ_th_ curve of the projected time period, whereas ${\hat{y}}_{\mathrm{n}+\upxi \left({x}_j\right)}$ shows the point projections for the observed sample [39].

### The multivariate Diebold–Mariano (DM) test

Diebold-Mariano (DM) test is the statistical test, introduced by Diebold and Mariano in 1995. This test is used to test the null hypothesis of equal forecast accuracy or equal predicting ability between two competing models. The DM test evaluation criteria is based on its parameter loss function. This loss function may include variety of differences and skill functions such as straight, absolute or squared differences and correlation respectively. In particular, the test is free from the distributional assumption on the forecast errors. It can includes temporal autocorrelations and any other type of correlation between the two or more series. Variety of alterations to the test have been prepared for its improvement and for conveniently to use it [40–43]. In order to test the forecast accuracy among multiple models, the authors Mariano and Preve extended the DM test for multivariate forecast accuracy [44]. The general loss is used to evaluate the multiple forecast models. The framework of multivariate DM test is describes as follows. The test consist of null hypothesis that demonstrates equal predictive accuracy for the predictive models. The test procedure is as follow:

9 $$ {H}_0:E\left[g\left({e}_{(1),t}\right)\right]=E\left[g\left({e}_{(2),t}\right)\right]=\dots =E\left[g\left({e}_{\left(k+1\right),t}\right)\right], $$

10 $$ {e}_{(i),t}=\left\{{y}_{it}-{\hat{y}}_{it}\ \right\},i=1,2,\dots, k+1.t=1,2,\dots, T $$

In the above Eq. (9) the component *E*[*g*(*e*_(1), *t*_)] indicate the loss function expected value for the i^th^ forecast. While in Eq. (10) the *e*_(*i*), *t*_ is the (k + 1)^th^ forecast errors time series from (k + 1) another model. In simple word, we can assume that these components might be the forecasts outcome that based on some subjective or objective approach, for example, surveys, judgments, extrapolation techniques, smoothing, time-series models, or any mixture of methods. The accuracy of the forecasts is to be assess using some specified loss function that is denoted by *g*(∙). As various parameter can be involved in the loss function, so usually it is assume that the loss function depends only on the forecast errors. The loss function differential *d*_*jt*_ between forecasting errors of two model is described as

11 $$ {d}_{jt}=g\left({e}_{j,t}\right)-g\left({e}_{j+1,t}\right),j=1,2,\dots, k $$

For multiple model, *k* loss differential series { *d*_*jt*_ } in notation of vector is considered as:

12 $$ {d}_t=\Big({d}_{1t,}{d}_{2t},\dots, {d}_{kt}\overset{\acute{\mkern6mu}}{\Big)},t=0,\pm 1,\pm 2,\dots $$

In context of Eq. (12), Now the null hypothesis of Eq. (9) can be stated as *H*_0_ : *E*[*d*_*t*_] = 0. In order to test this null hypothesis, the vector of observed sample means for equal predictive ability test is written as:

$$ \overline{d}=\frac{1}{T}{\sum}_{t=1}^T{d}_t $$

For example *d*_*t*_ is the ccc (AFE). So it can be expressed as:

13 $$ {d}_t=\mu +{\varepsilon}_t+{\theta}_1{\varepsilon}_{t-1}+{\theta}_2{\varepsilon}_{t-2}+\dots $$

Finally, according to above equation the multivariate version of the DM test statistic is designed as:

14 $$ S=T{\overline{d}}^t{\hat{\Omega}}^{-1}\overline{d}\to {\chi}_k^2 $$

where *T* is total number of years in the predictive set and transpose matrix of $\overline{d}$ is denoted as ${\overline{d}}^t$. While $\hat{\Omega}$ is the stable estimator of Ω and k is the degree of freedom that is number of time series in the system. Heteroscedasticity and autocorrelation consistent (HAC) estimate of Ω can provide improvement in the time related structure without distressing test statistic distribution [44, 45]. In addition, a study based on Monto Carlo simulation suggested that a reasonable estimator of Ω can also be attained using the sample variance [46].

Level of significance alpha (α) can be considered for decision of null hypothesis (H_0_). Rejection of null hypothesis suggests that not all tested model holds equal predictive ability. Hence, loss dissimilarities in $\overline{d}$ are consider for the choice of the outstanding predictive model.

## Results of empirical analysis

### Descriptive epidemiology

The annual four Asian countries mortality rates due to breast cancer (BC) from 1990 to 2017 for age range 20 to 80+ year was considered to run the application of three stochastic mortality models with smoothing p-splines approach. The data related to four Asian countries, China, Pakistan, India and Thailand was downloaded from the Institute for Health Metrics and Evaluation (IHME) http://ghdx.healthdata.org/gbd-results-tool. The accessibility of data and sources are mentioned in “availability of data and materials” statement at the end of the paper. We considered women BC mortality rates from 20 to 80+ years of age and for each calendar year 1990 to 2017 for four Asian countries. The mortality rates due to breast cancer given by the ratio between the “number of deaths” and the “exposure to risk,” were arranged in a matrix for specific age x and time t. In order to assess the differences in BC mortality rates corresponding to both age x and time t, we realized that BC mortality has shown a gradual increase with time. To have an idea of this progression, the general trends in the BC mortality rates and log mortality rates during the period 1990–2017 for four Asian countries are depicted in Fig. 1. We can see that mortality improvements are not similar across the ages and the years. As it is clear, there is an increasing difference for higher ages (> 50), particularly around age = 80 for each country except Thailand where slightly declining trends are observed with time.

**Fig. 2:**
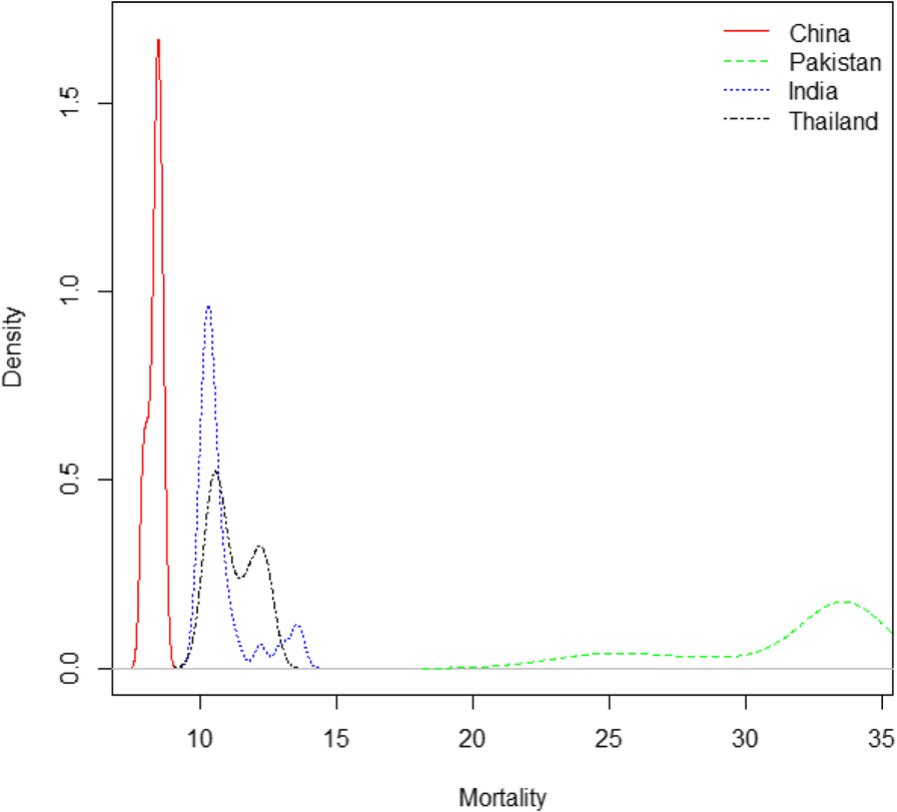
Density plots of females’ age-standardized mortality rates (per 100,000) due to breast cancer, by countries, from 1990 to 2017

Additionally, Density plot of age standardized BC mortality rates for four countries’ data sets are presented in Fig. 2. Rates are more dispersed in Pakistani women while India and Thailand data showed the monotonic differences with improvement in BC mortality.

### Model fits

In order to assess the in-sample and out-sample model performance, we fit the models by excluding the last 7 years of data from each country’s data set. The first step of analytical analysis is fitting the three stochastic mortality models after applying the smoothing p-splines approach for each country’s data sets under consideration. Which later called as, smoothed lee carter (SLC) model, Functional demographic model (FDM) and Booth-Maindonald-Smith (BMS) model. Supplementary Figs. S1, S2 and S3 show the estimated parameters from these three models. The percentage of variation (PV) explained by the SLC model is 97.3, 89.3, 63.7 and 87.8% for the China, Pakistan, India and Thailand data respectively. The difference among the PV of four countries’ data set is due to their BC mortality behavior and different features of data as shown in Figs. 1 and 2. We can see that the BC mortality rates at older ages in China data are more consistent than other countries; as a result, the SLC model fitted the China data better than the other country’s data set.

Moving from the SLC to the second stochastic mortality model that called as FDM, we notice that the PV explained by the FDM model increases for Pakistan and Thailand while decreases for India and remain almost constant for China. Specifically, in context of order 3 of FDM, the basis functions explain respectively 97.1, 1.9, 0.4% of the variation for China, 90.0, 8.7, 0.8% of the variation for Pakistan, 61.6, 20.5, 7.5% of the variation for India and 88.4, 10.2, 0.7% of the variation for Thailand data. The difference of variations among basis functions is because these basis functions model the different movements in BC mortality across the ages. Specifically, the first basis function usually models the movements in BC mortality for younger ages. Let consider the Figs. S1, S2 and S3 for SLC, BMS and FDM model respectively, to highlight some differences among four countries death rates due to breast cancer. Keeping in view the Fig. S3, we notice that the function fitted on China, Pakistan and Thailand data has a stronger slope between age 20 and 30, 20 and 40 and 30 and 40 respectively than the fitted function on India data. Therefore, the BC mortality reduction at younger ages is greater for China, Pakistan and Thailand comparatively to India. Correspondingly, the first fitted coefficient plot shows the evidence about the mortality improvements at younger age. We can observe that, the BC mortality rates for younger ages have dropped over the period 1990–2005 and this phenomenon is captured by the decreasing trend of the coefficient 1 for China and India. On the other hand, a gradually increasing trend of the coefficient 1 is observed over the whole period for Pakistan data. The second basis function provide us evidence about the BC mortality rates differences between 30 and 60 years old. It can be clearly observe that almost each country have more strained rate differences but this differences is more stressed for Pakistan data as compare to other countries. Finally, the function 3 represents patterns that are more complex and it model the BC mortality differences between all the cohorts. In the current situation, these differences are not much significant for each country under consideration. Therefore, third functions show a lower variability between ages 65 to 80. Further, shifting from the SLC and FDM to the BMS model, we observed that the PV explained by the model was consistent with the explained variation by SLC model. On the other hand, fitting from the both SLC and BMS model shows the higher variances in upper ages (> 50) for Pakistan than other Asian countries while with time these mortality differences are increases after 2005 period particularly for Pakistan and India (Figs. S1 and S2).

### Goodness of fits of models

#### Residual analysis

**Fig. 3:**
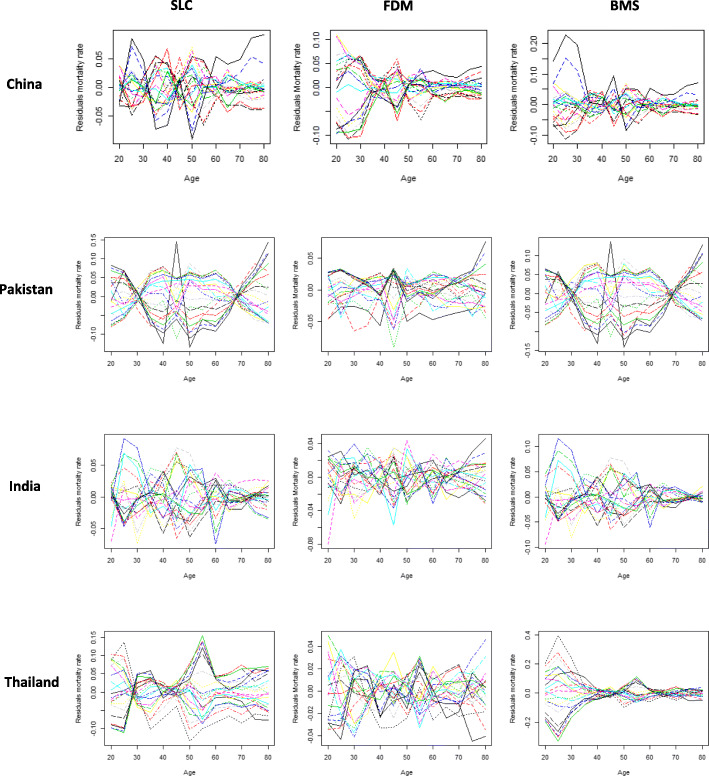
Residuals BC mortality rates from three models, SLC, FDM and BMS, for each country individually

A good fit is achieved when the residuals are independent and identically distributed. In order to verify this condition, residuals mortality rate by age in different years was derived from three fitted models for all four countries’ data set (Fig. 3). Residuals mortality rate by age were seem to be more consistent in FDM than in SLC and BMS for all four countries almost everywhere. Whereas, these errors were lower in SLC and BMS model on older ages for each country’s data set. In addition, the error measures were also calculated to conform the error differences among different models as shown in Table 1. By comparing the SLC, FDM and BMS model, we notice that overall the error measures in FDM are smaller as compare to other models, even if the PV described by model is greater in the both SLC and BMS as compare to FDM model. Specifically, the mean square error (MSE) of the SLC model is lower than the MSE of the other two model for China while for other three countries’s data set this error is the lower in FDM as compare to other two model.

**Table 1:** Error measures from three fitted models individually for each country

	**Smoothed Lee Carter (SLC) Model**			
**Average across ages**	**ME**	**MSE**	**MPE**	**MAPE**
China	0.000	0.0008	0.020	0.036
Pakistan	0.000	0.0024	0.0004	0.016
India	0.000	0.0007	0.0232	0.073
Thailand	0.000	0.0023	0.0110	0.056
**Average across years**				
China	0.000	0.046	1.509	2.736
Pakistan	0.000	0.134	0.018	0.794
India	0.000	0.0418	1.7075	5.827
Thailand	0.000	0.133	0.803	4.153
**Average across ages**	**Functional Demographic Model (FDM) **			
China	0.000	0.0013	−0.029	0.065
Pakistan	−0.000	0.0004	0.001	0.008
India	0.000	0.0003	0.0128	0.046
Thailand	−0.000	0.000	0.005	0.019
**Average across years**				
China	−0.000	0.0700	−2.199	4.815
Pakistan	−0.000	0.026	0.0437	0.367
India	−0.000	0.016	0.939	3.575
Thailand	−0.000	0.015	0.378	1.359
**Average across ages**	**Booth-Maindonald-Smith (BMS) Model**			
China	−0.000	0.0014	0.033	0.054
Pakistan	0.001	0.002	0.001	0.016
India	0.000	0.001	0.022	0.075
Thailand	−0.000	0.006	0.032	0.141
**Average across years**				
China	−0.007	0.084	2.466	4.109
Pakistan	0.077	0.139	0.048	0.788
India	−0.000	0.045	1.675	5.909
Thailand	−0.004	0.362	2.348	10.701

*ME* mean error, *MSE* mean square error; *MPE* mean percent error; *MAPE* mean absolute percent error

#### In-sample and out-sample predictive accuracy by multivariate Diebold-Mariano (DM) test

In order to test and compare the model predictive accuracy we used the multivariate Diebold-Mariano (DM) test, which is advance approach for testing and comparing of predictive ability of models. To measure in-sample forecast accuracy, the multivariate DM test is applied to average Absolute Forecast Error (AFE) across ages and years from three models for each country’s data set. According to the test, we have formulated our null hypothesis (H_0_) that “predictive accuracy of the three model is the same” that implies zero difference of average absolute forecast errors among competing models. Based on our results in Table 2, the null hypothesis is not accepted at 1% level of significance for all four countries results (*p* < 0.01). Therefore, based on the results we may conclude that three models hold different predictive accuracy across years for each country’s data set. On the other hand, Table 3 test the predictive accuracy of three models across ages we observe that three models hold equal predictive accuracy across ages.

**Table 2:** Comparison of predictive accuracy of three models across years for each country using multivariate Diebold-Mariano (DM) test

Country	Null Hypothesis	Test Statistic Value	***P***-Value	Decision
China	H_0_: SLC=FDM = BMS OR H_0_: All model hold Equal predictive accuracy	− 1796.2	0.000	H_0_ is rejected
Pakistan		−20.151	0.000	H_0_ is rejected
India		−24.314	0.000	H_0_ is rejected
Thailand		−3.9457	0.000	H_0_ is rejected

Predictive period, 1990–2010

**Table 3:** Comparison of predictive accuracy of three models across ages for each country using multivariate Diebold-Mariano (DM) test

Country	Null Hypothesis	Test Statistic Value	***P***-Value	Decision
China	H_0_: SLC=FDM = BMS OR H_0_: All model hold Equal predictive accuracy	14.165	0.999	H_0_ is not rejected
Pakistan		2617.9	1	H_0_ is not rejected
India		1034.7	1	H_0_ is not rejected
Thailand		89.218	1	H_0_ is not rejected

Predictive period, 1990–2010

**Fig. 4:**
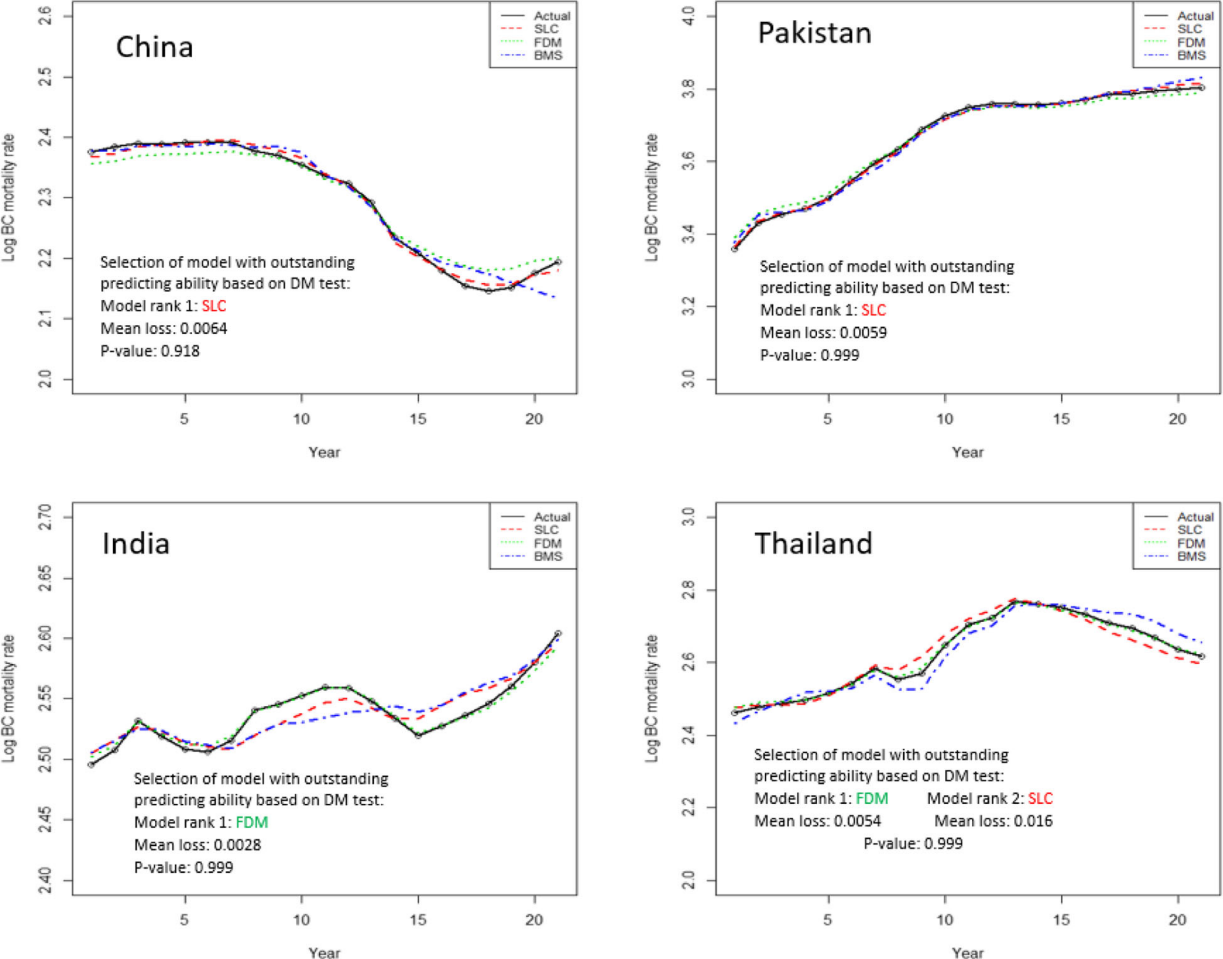
In-sample goodness of fit of three models and selection of best fit by multivariate DM test on four countries’ data set, models are fitted for 21 year of BC mortality rates from 1990 to 2010, values on x-axis showing the number of years from 1990 to 2010 with 5 year interval

In addition, we also test the model for outstanding predicting ability based on multivariate DM goodness of fit test. The DM test assigned rank 1 to the model which have minimum mean loss value among the models which indicating best fit of that model. According to the test, null hypothesis indicate the best fit of ranked 1 model. So, Fig. 4 depicts the actual and fitted log BC mortality rates across years from three models. We can see that according to multivariate DM goodness of fit test, the SLC model is ranked 1 which indicating best fit of model for China (Mean loss = 0.0064, *p*-value = 0.918) and Pakistan (Mean loss = 0.0059, *p*-value = 0.999). On the other hand, FDM model showed best fit on India (Mean loss = 0.0028, *p*-value = 0.999) and Thailand (Mean loss = 0.0054, *p*-value = 0.999) data.

In order to compare the in-sample predictive accuracy among three models, we demonstrated that SLC model provided the outstanding predictive ability for China and Pakistan data set while FDM delivered the outstanding predicting ability on India and Thailand data.

**Fig. 5:**
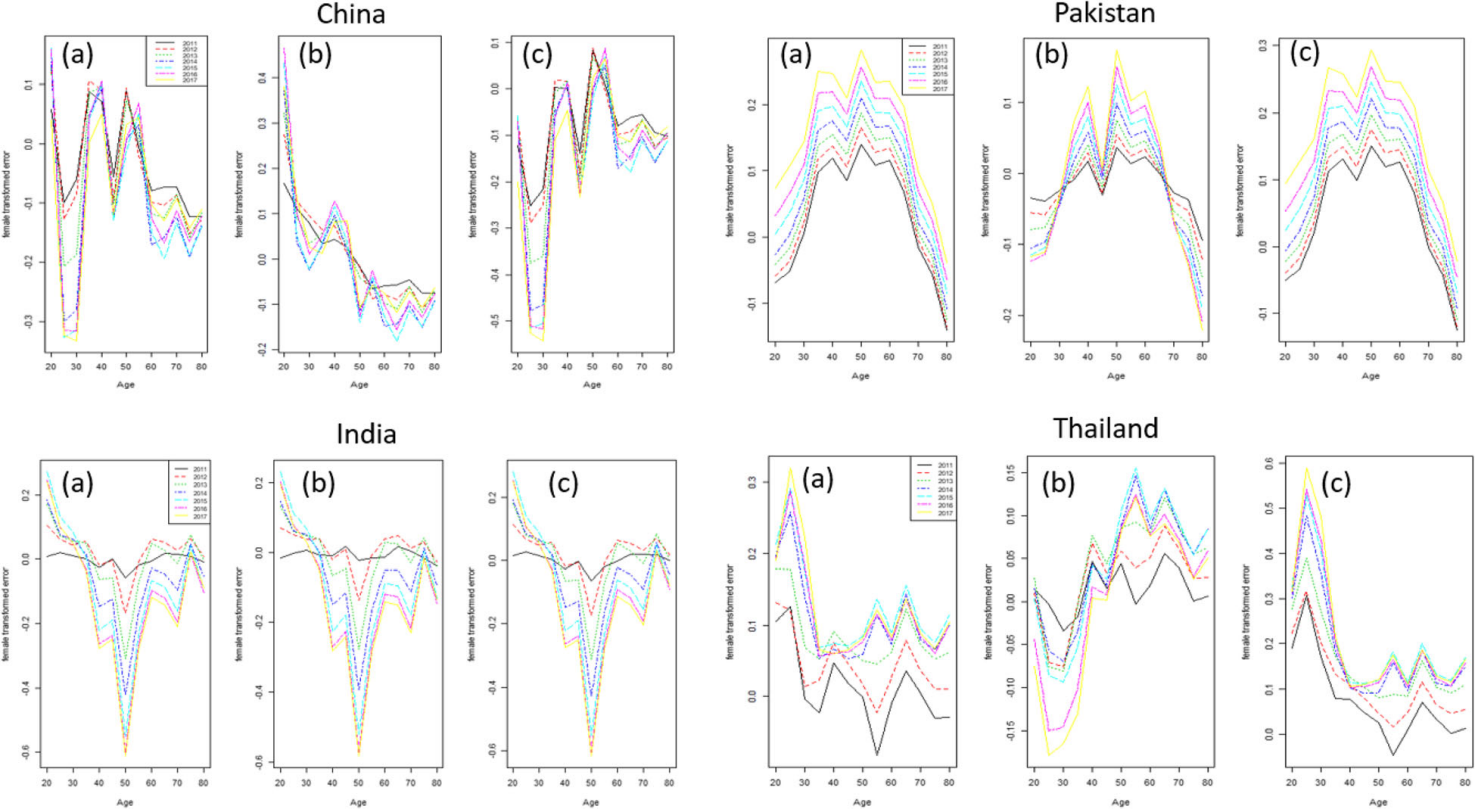
Forecast errors of **(a)** SLC **(b)** FDM **(c)** BMS model across ages by forecast year 2011–2017 for four countries’ data

**Fig. 6:**
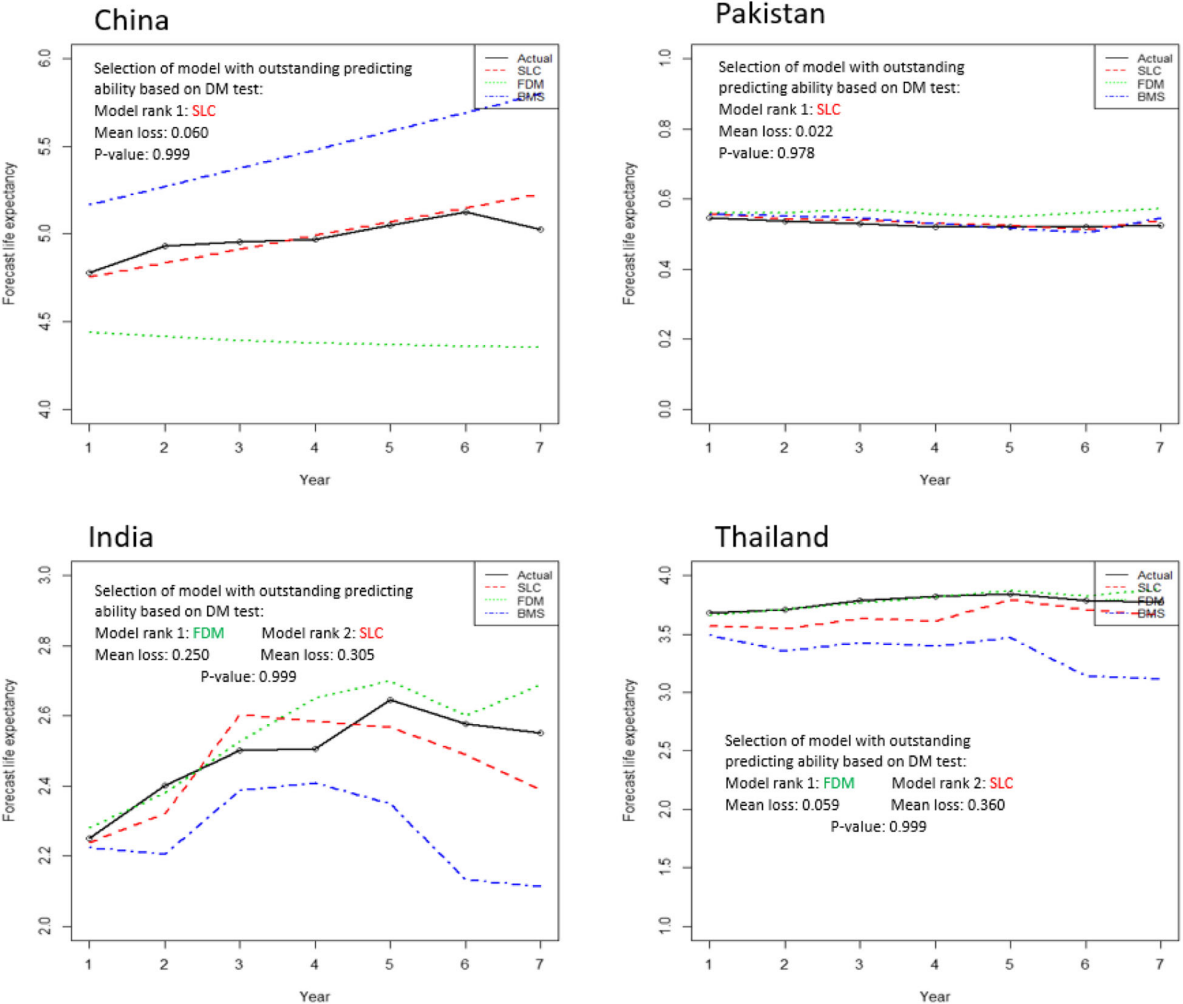
Out-sample goodness of fit of three models and selection of best fit by multivariate DM test on four countries’ data set, 7 year forecast from 2011 to 2017 are compared among the models, values on x-axis representing the code of years from 2011 to 2017

In order to conform the forecast ability of models, it was also tested, the model that provide a good in-sample fit to historical data whether it still produce good out-sample forecasts. A good model should provide accurate in-sample fits to the historical data as well as out-sample plausible forecasts. Therefore, out-sample predictive accuracy was also considered to verify the model predictive accuracy consistency. To evaluate the forecast accuracy, the following steps are required. Initially, it is needed to choose the matric of interest included the forecasted variable. Possible metrics of forecasted variables may include the mortality rates, life expectancy or future survival rates. Different metrics are relevant for different purposes. As, our study goal is to examine the feasibility of different stochastic mortality models, hence, we emphasis on the BC mortality rates. We project the BC mortality rates from 2011 to 2017 according to the fitted models and derive the life expectancy to compare the forecasts with the actual. In our study forecasts are calculated based on the evolution of time parameter (*k*_*t*_) and errors in age parameter (*a*_*x*_) and (*b*_*x*_) are not considered. Because according to the literature the standard errors of (*a*_*x*_) and (*b*_*x*_) to become less significant over forecast time in comparison to the standard error of parameter (*k*_*t*_) [11]. The forecast errors in the SLC, FDM and BMS model for four countries’ data set are shown in Fig. 5. To compare the out-sample forecast accuracy among three models, average projection error in life expectancy by computing mean over forecast years were considered. Table 4 displays the comparison of out-sample forecast accuracy based on forecast errors in life expectancy. Based on multivariate DM test results we determine that three models have significantly different (*p* < 0.01) out-sample predictive accuracy for all countries’ data set (Table 4). Moreover, we also find the model with outstanding forecast ability for four countries’ data set. According to the test results, SLC model provided the outstanding forecast ability on china and Pakistan data, while FDM has rank 1 for providing outstanding forecast ability for India and Thailand data set. At the same time, SLC model also offered the quite comparable results for India and Thailand data (Fig. 6).

**Table 4:** Comparison of forecast accuracy of three models across years for each country using multivariate Diebold-Mariano (DM) test

Country	Null Hypothesis	Test Statistic Value	***P***-Value	Decision
China	H_0_: SLC=FDM = BMS OR H_0_: All model hold Equal predictive accuracy	64.354	0.000	H_0_ is rejected
Pakistan		7.636	0.000	H_0_ is rejected
India		3624.1	0.000	H_0_ is rejected
Thailand		286.68	0.000	H_0_ is rejected

Forecast period, 2011–2017

**Fig. 7:**
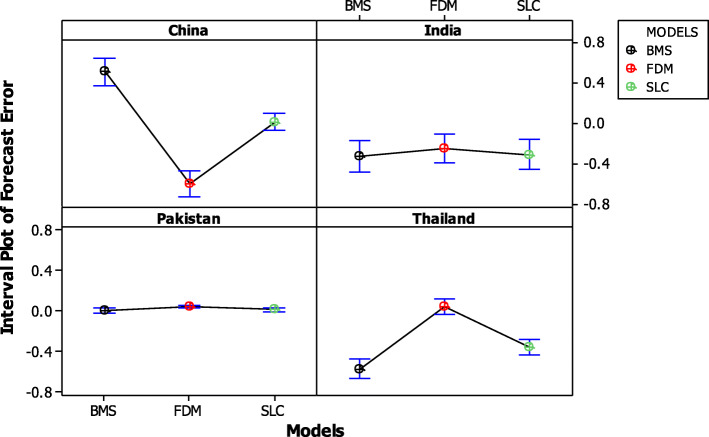
Confidence Interval (95%) for the mean forecast error of SLC, FDM and BMS model separately for each country’s data set

Further, we also calculate the mean and variance of life expectancy forecast errors across forecast years. Table 5 depicts the minimum variance of life expectancy forecast error for SLC model in China and Pakistan data set. On the other hand, the FDM and SLC model showed the almost same variance for India and Thailand data. Moreover, 95% confidence interval plot of BC mortality forecast error were also shown in Fig. 7. The confidence intervals for the mean error are tighter for SLC model than other models in both China and Pakistan data set. However, the confidence intervals for the both model FDM and SLC are narrow for other two countries’ data set.

**Table 5:** Mean and variance of forecast error in life expectancy

Country	SLC		FDM		BMS	
	Mean	Variance	Mean	Variance	Mean	Variance
China	0.015969	0.007437	−0.58973	0.01647	0.507574	0.017841
Pakistan	0.012938	0.000478	0.044085	0.000288	0.003414	0.000489
India	−0.30533	0.023026	−0.24113	0.020099	−0.32129	0.023243
Thailand	−0.36042	0.006641	0.044443	0.006649	−0.57447	0.010475

## Discussion

Although, the forecasting mortality methods have been broadly studied by a group of researchers like Booth [22] and Booth & Tickle [23]. In the present study, we apply the three stochastic mortality models belong to family of generalized lee-carter with smoothing p-splines approach to four Asian countries’ data sets, evaluate the performance of these three methods, compare the similarities of different Asian regions and propose potential improvements. Furthermore, study is also contain the derivation of future stochastic life expectancy at birth calculated from mortality forecasts and use it for models assessment.

The age-specific BC mortality change by different years for four Asian countries’ data set was depicted in Fig. 1. We can observe that, the mortality rates has followed a smooth function with some observational error. The observational error has higher variance at very old ages where the population are small and also at young ages where the mortality rates are small. This phenomenon can also be clearly shown from Fig. 2 that have higher intensity of mortality for small population of Pakistan while remaining countries have relatively smaller mortality rates for large number of population. A study also reported the similar pattern in USA mortality data, where researchers observed that age-specific mortality rates are higher than 1/100,000 for very small populations [38]. Mortality forecasting based on such a data pattern may be well representative by stochastic mortality models with P-splines smoothing approach. This smoothing approach have been extensively used to smooth mortality data for application of these models in various studies [10, 26, 27, 38].

Application of stochastic smoothed mortality models namely SLC, FDM and BMS model on BC mortality data provided the different PV explained by the models. Consistent PV among three models were observed for China data set. The reason may be the method assume the homoscedastic variances as this data is follow to this assumption to some extent [38]. The other reason might be the China’s data sets contain less wiggly data in a shorter and older age range. While for other countries these PV were little bit different among the three models, which shows the capability of the models to deal with the heteroscedastic data. It observed that for these type of data FDM provided the larger PV and smaller measure error than the other models. In line with previous literature, the basis functions used in FDM model have the ability to account different mortality movements and model them across the ages [15]. Therefore, larger PV explained by FDM in irregular data set might be the reason of presence of different basis function in the model.

To highlight some differences among four countries’ death rates due to breast cancer we fitted the three models on each country’s data set separately (supplementary Figs. S1, S2 and S3). Keeping in view the FDM results (Fig. S3), we observed that the fitted mortality functions, from age 20 to 30 for China, 20 to 40 for Pakistan and 30 to 40 for Thailand has a stronger negative slope. Therefore, the BC mortality reduction at younger ages was greater for these countries as compare to India. The decreasing trend in BC mortality in these countries has been reported previously and reason of this decline may be due to higher pregnancy rates and related reproductive factors in that countries [47, 48]. In addition, low or never use of oral contraceptives may also be the possible reason for the decreasing BC mortality rates in younger women [4, 49]. Both SLC and BMS model showed the higher mortality variances in upper ages (> 50) for Pakistan than other Asian countries during the study period. Mortality differences gradually improved after 2005 period in Pakistan and India and remained consistent or move to decline in China and Thailand during the specified time period. The actual cause of the reduction in breast cancer mortality is still unknown yet and required more research to explore the possibility. Proper Access to medical facilitates and advanced treatments are likely reasons for the decline in mortality in China: such as improved early detection combined with effective treatment. Most women under 50 years of age working in urban areas have employer-sponsored benefits like medical examinations and free breast ultrasounds once or twice a year. Previous studies have shown that ultrasound is superior to Chinese women’s mammography for the prevention and control of BC [50]. Mubarik et al. [4, 7] conducted a study on trends and projections in BC mortality, and they reported the higher BC mortality rates during the period 1990–2017 in some of Asian countries including Pakistan and India. Our study results has also reported the similar findings in these countries. The rising trends in BC mortality in these countries might be due to that they did not have much success in disease diagnosis and treatment programs as compare to developed Asian and European countries during the developmental period.

In order to evaluate the models performance, both in their goodness of fit on historical data and their forecasting ability (in-sample and out-sample), all models were tested and ranked by using residual analysis and an advance statistical multivariate DM test. This test is widely used for measuring predictive ability across multiple forecasting methods [51]. According to goodness of fit analysis, FDM errors were generally lower and residual trends were more consistent. In addition, the error measures suggested by Cairns et al. [18] were also computed and compared for the three models. We noticed that overall the error measures in FDM were smaller as compare to other models, even if the PV explained by model was higher in the SLC and BMS than in FDM. Specifically, the MSE of the FDM model was lower than the MSE of the other two model for each country’s data set except China. Furthermore, multivariate DM test had showed the significant difference among the models predictive performance for each country’s data set. When outstanding forecasting ability of models was tested for both in-sample and out-sample forecasts, SLC model showed the best predicting ability in both cases for China (in-sample mean loss: 0.006, *p* = 0.918; out-sample mean loss: 0.060, *p* = 0.999) and Pakistan (in-sample mean loss: 0.005, *p* = 0.999; out-sample mean loss: 0.020, *p* = 0.978). While FDM model indicated the best predictive performance within and outside forecasts for data set of other two countries. In contrast, model choice based on goodness of fit testing was ambiguous. Overall, study results indicated that the model/models, which had similar predictive ability within data, they also provided the almost same predictive ability of outside of fitted data. Nevertheless, there was no single model that we called the overall best model for all countries’ data set. Overall, based on model predictive accuracy tests, SLC model generally outperform the FDM for China and Pakistan data set, while FDM was ranked 1 on India and Thailand data both on goodness of fit and on forecast accuracy. These findings are consistent with the claim by other studies that have reported their findings in favor of the FDM model than the Lee-Carter model in term of better performance [10, 52].

In order to confirm the predictive accuracy results, we also calculated the mean and variance of forecasting errors in life expectancy over forecast years. The minimum mean error and variance of forecast error in life expectancy for SLC model for China and Pakistan data was confirming the predicted accuracy of that model. On the other hand, the FDM and SLC model showed the almost same variance for India and Thailand data. Moreover, 95% confidence interval plot of BC mortality forecast error were also shown. The confidence intervals for the mean error was tighter for both SLC model and FDM for Pakistan and Thailand. A quantitative comparison study of different stochastic mortality models has also reported the consistency in performance of lee-carter and functional demographic models on Italian mortality data [19].

## Conclusion

According to the study findings, we conclude that in selecting and ranking the mortality models, they should carried out for both their goodness of fit and testing of within and out data forecasting ability. In particular, application on BC mortality data under study, stochastic smoothed mortality models based on functional data analysis, generally had better perform on quadratic structure of data, both in term of goodness of fit and on forecast accuracy. Commonly, their errors were lower and their error distributions although were not ideally satisfying but showed less inconsistent effects. In some of previous studies, application of these models on mortality data also claim in favor of FDM that it outperform the lee-carter model [10, 18]. On the other hand, SLC model outperform the FDM in case of symmetric data, and also shows almost comparable results to FDM for both within and outside data forecast accuracy in case of asymmetric or quadratic structure of data.

Therefore, our study recommends considering the SLC model in comparison to the other, since it provides even better results in some cases. Moreover, each model considered has certain limitations and shows certain failures to present all data. Therefore, no one can eventually be convinced of “the finest” in fitting and predicting BC mortalities. Although the results of both models, FDM and SLC included, were generally fair. However, they still display some nonrandom errors because of the cohort effect. Therefore, we plan to consider and compare other stochastic mortality models with cohort effect in future work and test them on smoothed BC mortality data. In conclusion, as already renowned by other studies [19, 52] we also suggest generating mortality forecasts using multiple models rather than relying upon any single model. In the current case, we can assume that there is no single model, which can truly outperform all the others on every population. Additionally, we perceived that these approaches would be most convenient for modeling and projecting the future trends of rare diseases for which there has been very little improvement in treatment and involve least birth cohort effects.

## Supplementary Information

**Additional file 1: Figure S1.** The parameter estimates of SLC model for four countries’ BC mortality rates, ax, is the derived age pattern averaged across years; bx, stands for the sensitivity of the mortality rates to the change of kt, reflecting how fast the mortality rate changes over ages; kt represents the only time-varying index of mortality level. **Figure S2.** The parameter estimates of BMS model for four countries’ BC mortality rates, ax, is the derived age pattern averaged across years; bx, stands for the sensitivity of the mortality rates to the change of kt, reflecting how fast the mortality rate changes over ages; kt represents the only time-varying index of mortality level. **Figure S3.** The parameter estimates of FDM model on four countries’ BC mortality rates, (a) China (b) Pakistan (c) India and (d) Thailand.

## Data Availability

The dataset analyzed during the current study are available in the Institute for Health Metrics and Evaluation (IHME): http://ghdx.healthdata.org/gbd-results-tool. Statistical analysis was performed using the R demography and forecast evaluation package, which codes are available on GitHub (https://github.com/robjhyndman/demography); (https://github.com/johntwk/Diebold-Mariano-Test).

## Abbreviations

BC: Breast cancer
LC: Lee-carter
SLC: Smoothed lee carter
FDM: Functional demographic model
SVD: Singular value decomposition
BMS: Booth-Maindonald-Smith
DM: Diebold–Mariano
ARIMA: Autoregressive integrated moving average
MSE: Mean square error
AFE: Absolute forecast error
