# Supplementary material for: A multi-country comparison of stochastic models of breast cancer mortality with P-splines smoothing approach
Source: BMC Med Res Methodol. 2020 Dec 9;20:299. doi: 10.1186/s12874-020-01187-5 (PMC7727112; doi:10.1186/s12874-020-01187-5)
Supplement: Supplementary file 1 — Additional file 1: Figure S1. The parameter estimates of SLC model for four countries’ BC mortality rates, ax, is the derived age pattern averaged across years; bx, stands for the sensitivity of the mortality rates to the change of kt, reflecting how fast the mortality rate changes over ages; kt represents the only time-varying index of mortality level. Figure S2. The parameter estimates of BMS model for four countries’ BC mortality rates, ax, is the derived age pattern averaged across years; bx, stands for the sensitivity of the mortality rates to the change of kt, reflecting how fast the mortality rate changes over ages; kt represents the only time-varying index of mortality level. Figure S3. The parameter estimates of FDM model on four countries’ BC mortality rates, (a) China (b) Pakistan (c) India and (d) Thailand. [file 12874_2020_1187_MOESM1_ESM.pdf]

A multi-country comparison of stochastic models of breast cancer mortality with P-splines smoothing approach

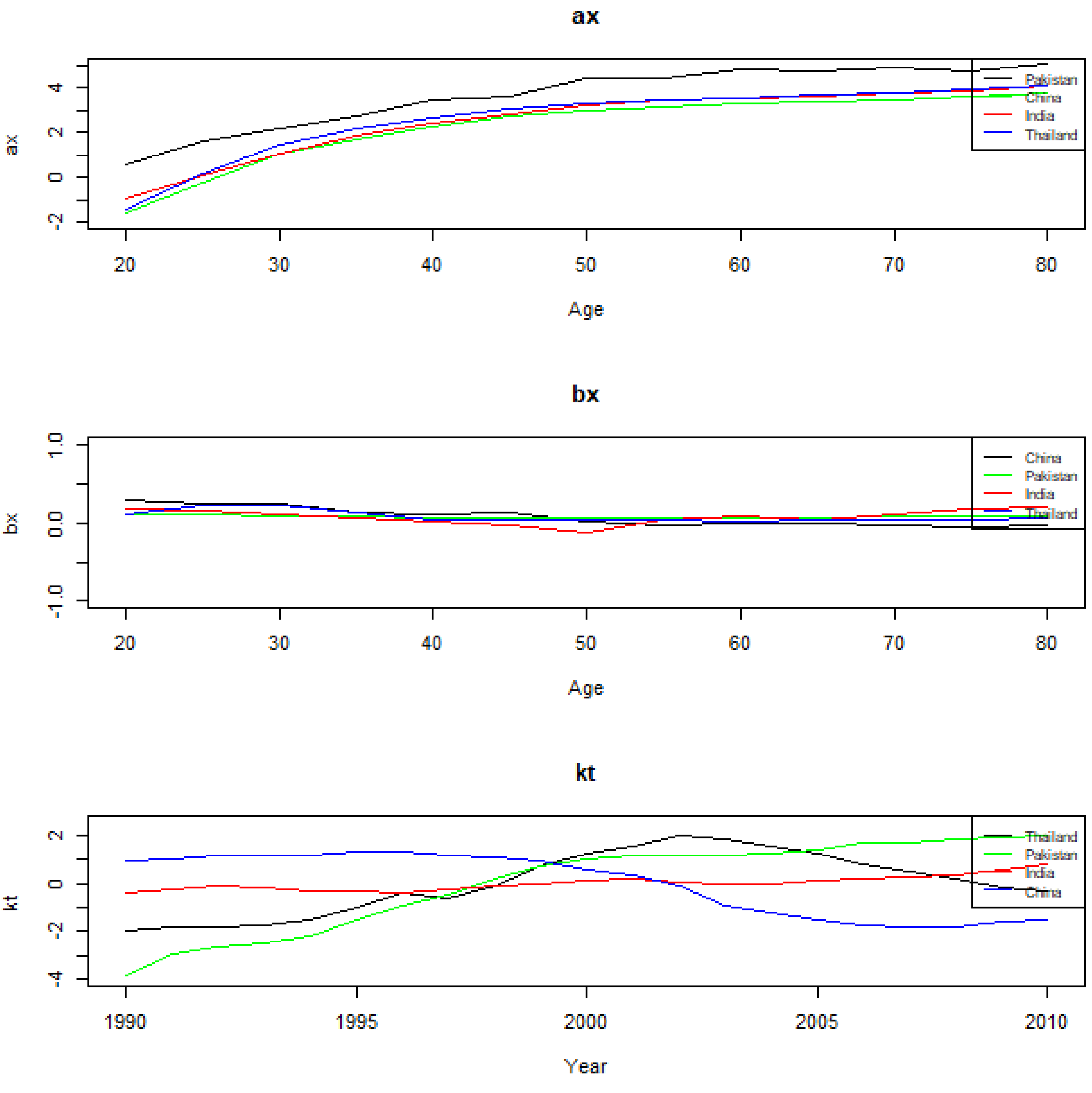

Figure S1: The parameter estimates of SLC model for four countries' BC mortality rates, **ax,** is the derived age pattern averaged across years; **bx,** stands for the sensitivity of the mortality rates to the change of kt, reflecting how fast the mortality rate changes over ages; **kt** represents the only time-varying index of mortality level

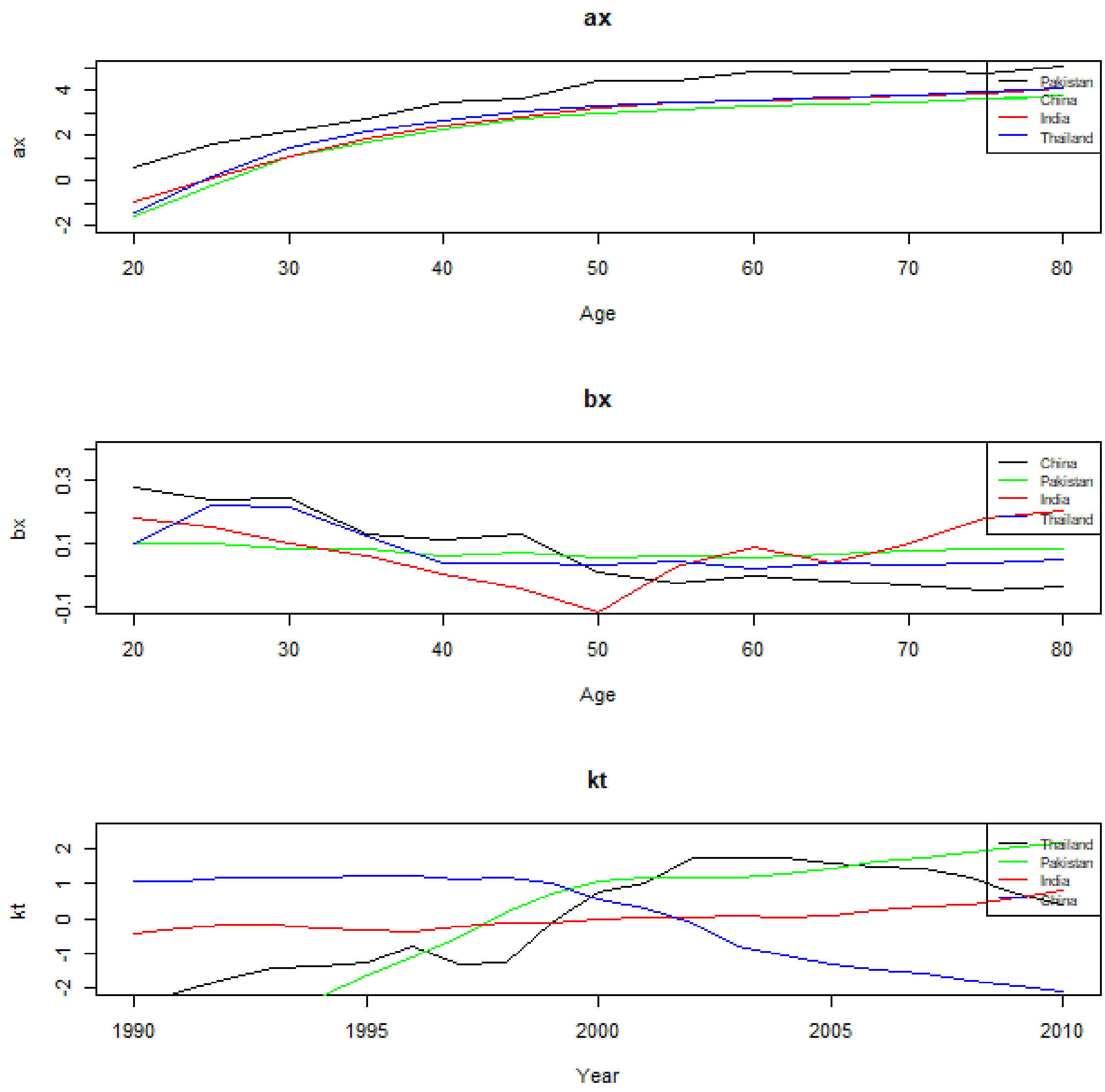

Figure S2: The parameter estimates of BMS model for four countries' BC mortality rates, **ax,** is the derived age pattern averaged across years; **bx,** stands for the sensitivity of the mortality rates to the change of kt, reflecting how fast the mortality rate changes over ages; **kt** represents the only time-varying index of mortality level

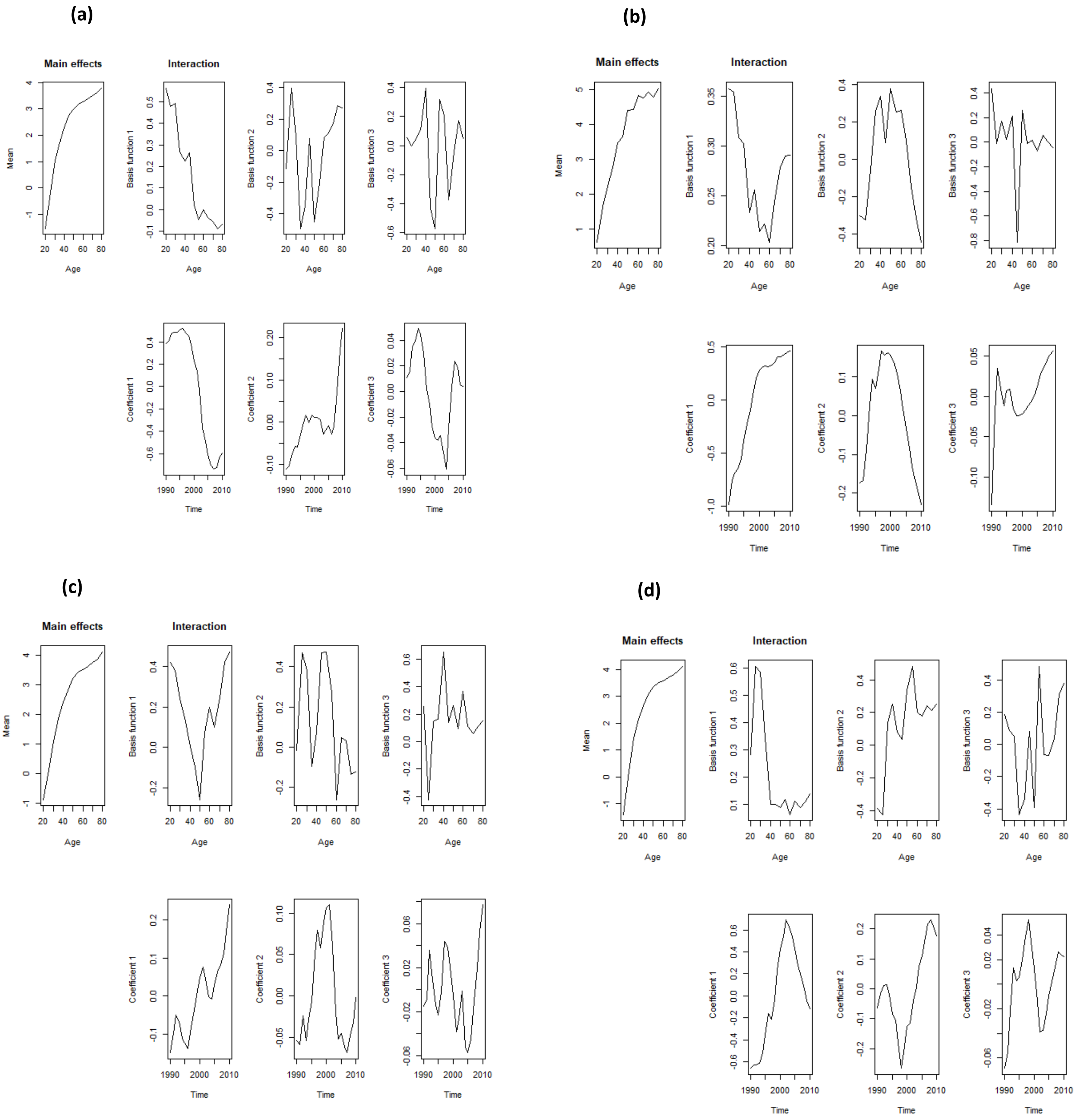

Figure S3: The parameter estimates of FDM model on four countries' BC mortality rates, **(a)** China **(b)** Pakistan **(c)** India and **(d)** Thailand
